# The impact of liver resection on survival outcomes of hepatocellular carcinoma patients with extrahepatic metastases: A propensity score matching study

**DOI:** 10.1002/cam4.1738

**Published:** 2018-08-16

**Authors:** Kai Mao, Yongcong Yan, Jianlong Zhang, Jie Wang, Ruomei Wang, Xiaojuan Ling, Yingyue Liu, Wan Yee Lau, Shuai Jiang, Jieqiong Liu, Zhiyu Xiao

**Affiliations:** ^1^ Guangdong Provincial Key Laboratory of Malignant Tumor Epigenetics and Gene Regulation Department of Hepatobiliary Surgery Sun Yat‐sen Memorial Hospital Sun Yat‐sen University Guangzhou China; ^2^ Faculty of Medicine Prince of Wales Hospital The Chinese University of Hong Kong Shatin Hong Kong SAR China; ^3^ Department of Epidemiology Johns Hopkins Bloomberg School of Public Health Baltimore Maryland; ^4^ Guangdong Provincial Key Laboratory of Malignant Tumor Epigenetics and Gene Regulation Breast Tumor Center Sun Yat‐sen Memorial Hospital Sun Yat‐sen University Guangzhou China

**Keywords:** extrahepatic metastasis, hepatocellular carcinoma, primary tumor resection, propensity score matching, surveillance epidemiology and end results database

## Abstract

**Background:**

The majority of hepatocellular carcinoma patients (HCCs) with extrahepatic metastases die of progressive intrahepatic tumor. There have been little data on the role of primary tumor resection (PTR) for HCCs with extrahepatic metastases but with resectable primary tumors.

**Methods:**

A retrospective study was conducted on HCCs with extrahepatic metastases with resectable primary tumors who either underwent or did not undergo PTR in the SEER registry between 2004 and 2013. The overall and cancer‐specific survivals (OS and CSS) were assessed by the log‐rank test and the Cox proportional hazard regression model. A propensity score matching was conducted to minimize biases. Validation was performed in another cohort from the Sun Yat‐sen Memorial Hospital (SYSMH).

**Results:**

Of the 529 HCCs with extrahepatic metastases with resectable primary tumors included into this study, 230 patients underwent PTR and 299 did not. The percentages of patients who underwent PTR increased from 38.6% in 2004 to 70.3% in 2013. In the propensity score‐matched patients, PTR was associated with improved OS (HR 0.310, *P *<* *0.001) and CSS (HR 0.326, *P *<0.001). These improvements in survivals remained significant after sensitivity analyses using multiple imputation. In the validation cohort from the SYSMH (n = 131), PTR was also correlated with improved OS (HR 0.508, *P *=* *0.002) and CSS (HR 0.568, *P *=* *0.017).

**Conclusions:**

This study using propensity score matching and multiple imputation demonstrated that PTR had a favorable impact on the prognosis of HCCs with extrahepatic metastases with resectable primary tumors. Further prospective randomized trials are needed to confirm these findings.

## 1 INTRODUCTION

HCC with extrahepatic metastases is in an advanced stage of the disease. The 7th American Joint Committee on Cancer (AJCC) puts HCCs with regional lymph node metastases and distant metastases at Stage IV of the disease. Except for the rare occasions that the primary tumor together with a solitary extrahepatic metastasis that can be resected, the prognosis is poor.^1^ Sorafenib is currently considered the standard treatment.^2^, ^3^ The majority (80%‐95.7%) of these patients die of progressive intrahepatic tumor leading to hepatic failure, but not extrahepatic metastases.^4^, ^5^, ^6^, ^7^ Previous studies using transarterial chemoembolization (TACE) or radiofrequency ablation (RFA) in HCC patients with extrahepatic metastasis which effectively controlled the primary tumors resulted in survival benefit.^6^, ^7^, ^8^ To the best of our knowledge, there have been no data on the use of primary tumor resection (PTR) for Stage IV HCC patients with resectable primary tumor. According to the National Comprehensive Cancer Network (NCCN) guidelines, hepatic resection is a curative treatment option for patients with adequate liver function (Child‐Pugh class A and some Child‐Pugh class B patients without portal hypertension), adequate liver remnant volumes, and a solitary HCC without major vascular invasion.^9^, ^10^ The presence of extrahepatic metastasis is a contraindication for hepatic resection.

A recent study revealed that locoregional treatments specifically targeting intrahepatic lesions, including surgical resection, RFA, TACE, or radiotherapy to be an independent favorable prognostic factor of long‐term survival (HR 0.591; 95% CI 0.436‐0.803; *P *=* *0.001).^6^ However, this study can be criticized because it is retrospective, had a small sample size, with technical limitations and selection biases.

In this study, we assessed the overall and cancer‐specific survivals of PTR in HCC patients with extrahepatic metastases. Liver resection is one of the locoregional methods that can be used to effectively control the primary tumor. It has its own limitations and indications when compared with TACE, RFA, and radiotherapy. Data were obtained from the SEER registry over a 10‐year period. Potential selection biases were minimized using multiple imputation and propensity score matching. The survival effect of PTR was validated using an independent patient cohort from the Sun Yat‐sen Memorial Hospital in China.

## 2 METHODS

### 2.1 Patients and data collection

The SEER program of the National Cancer Institute is the largest publicly available cancer dataset and provides cancer incidence and survival data from population‐based cancer registries. The SEER 18 registry database (1973‐2013) covering approximately 27.8% of the US population was used as the data source for this study. The data were coded and reported according to nationally established protocols coordinated under the auspices of the North American Association of Central Cancer Registries (NAACCR). A total of 63 513 liver cancer patients between 2004 and 2013 were identified. Patients were limited to those who presented with Stage IV (including lymph node metastasis and distant metastasis; AJCC 7th TNM Stage) and were recommended surgery. Exclusion criteria included (a) diagnosis at autopsy, (b) diagnosis by death certificates only, (c) surgery not recommended, (d) age <20 years, (e) patients who died or were lost to follow‐up within 1 month after diagnosis, (f) unknown TNM stage, and (g) prior malignancies at the time of liver cancer diagnosis. This resulted in a cohort of 529 patients for the final analysis.

To validate the findings from the SEER dataset, we retrospectively analyzed 131 HCC patients diagnosed with extrahepatic metastases between 1 January 2006 and 31 December 2015 at the Sun Yat‐sen Memorial Hospital. These patients were clinically or histopathologically diagnosed as hepatocellular carcinoma with lymph node metastases or distant metastases. The primary tumors were resectable, and these patients were recommended PTR. The resectability criteria of primary tumor include (a) adequate liver function (Child‐Pugh Class A or B), (b) solitary mass, or multifocal disease with less than 3 nodules which are located in one segment or one lobe of liver, (c) no major vascular invasion, (d) primary tumor with major vascular tumor thrombi, the involved vein or bile duct can be resected simultaneously, or the tumor thrombi can be removed completely from the vein or bile duct, (e) adequate future liver remnant (FLR; at least 20% without cirrhosis and at least 30%‐40% with Child‐Pugh Class A cirrhosis, adequate vascular, and biliary inflow/outflow).

The eligible patients were grouped according to whether or not they had undergone PTR. The primary endpoints included overall survival (OS), which was defined as the time from diagnosis to death of any cause or to the latest date of follow‐up and cancer‐specific survival (CSS), which was calculated from the date of diagnosis to the date of liver cancer‐related death or to the latest date of follow‐up. The data from the SEER Registry and the Sun Yat‐sen Memorial Hospital were rendered anonymous. Consent was obtained from the Ethics and the Medicine Institutional Review Board of the Sun Yat‐sen Memorial Hospital to conduct this study.

### 2.2 Statistical analysis

#### 2.2.1 Primary analysis

The clinicopathological and demographic data between the two groups were compared using the chi‐squared test. The survival curves were plotted by the Kaplan‐Meier method, and the differences were analyzed using the log‐rank test. Multivariate survival analyses were carried out using the Cox proportional hazard model, and the hazard ratios (HR) with 95% confidence interval (95% CI) were calculated. The Schoenfeld's global test was used to test the proportional hazards assumption of the Cox model. For the covariates which did not fit the proportional hazards assumption, the stratified Cox regression model was used. The Akaike information criterion (AIC) and the Bayesian information criterion (BIC) were calculated to select the best regression model.

#### 2.2.2 Sensitivity analysis using imputation and propensity score

In the SEER database, missing data of some of the key variables (eg, tumor size) could result in biases. Thus, a multiple imputation procedure by chained equations^11^, ^12^, ^13^ was applied to account for the missing values of these variables (we assumed unknown tumor size, AFP, primary tumor number, vascular invasion, extrahepatic extension, and radiotherapy were missing at random). A probabilistic rule, based on the regression models for each covariable with the other covariables serving as predictors, was used to impute possible values for the individual missing values. A full dataset was created after imputing for 10 times using the “complete” function in the MICE package.^13^, ^14^ After multiple imputation, propensity score‐based sensitivity analysis was done to minimize selection bias or a lack of covariate balance. For the SEER cohort, we performed logistic regression to select demographic and clinicopathological variables associated with the implementation of PTR. All variables with a univariate *P* value ≤0.20 were eligible for inclusion in the logistic regression model. The final multivariate logistic model was used to calculate the propensity score for each individual, which was the probability of the patient being treated with PTR. Patients who underwent PTR were matched to patients who did not undergo PTR by a propensity score ±0.5 in a 1:1 ratio. The quality of the matching was checked by calculating the standardized difference for each covariate. Univariate and/or multivariate survival analyses were performed in the propensity score‐matched populations using the same methods as those in the primary analysis.

Statistical analyses were conducted using STATA 12.0 software (StataCrop, College Station, TX) or R software (R Core Team 2014^15^). All statistical tests were two‐sided, and statistical significance was defined as *P *<* *0.05.

## 3 RESULTS

### 3.1 Clinicopathological characteristics of patients

**Figure 1:**
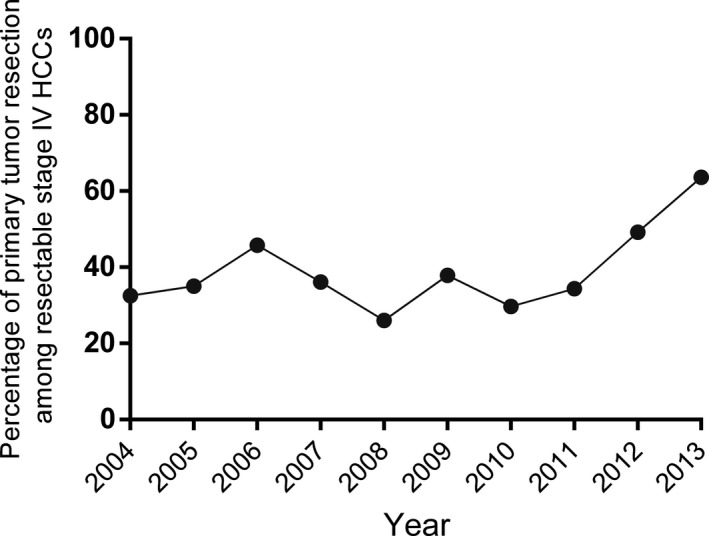
Trend of the rate of PTR in HCC patients with resectable primary tumor but with extrahepatic metastases in the SEER population between 2004 and 2013

Of the 529 HCC patients with extrahepatic metastases but with resectable primary tumors in the study, 230 patients underwent primary tumor resection, whereas 299 did not. The comparison of the clinicopathological characteristics of the patients who underwent PTR or not is outlined in Table 1. There were significant differences (*P* < 0.01) in the majority of the characteristics between the PTR group and the non‐PTR group, demonstrating that any comparisons between these two groups of patients would be influenced by selection biases. When compared with patients without PTR, patients who underwent PTR were younger, had huge tumors with diameter ≥10 cm, had single primary lesions, and more extrahepatic extension. There was an increasing trend in the rate of PTR in the SEER population in 10 years (Figure 1). The rate of PTR increased from 38.6% in 2004 to 70.3% in 2013 (*P *<* *0.001), although it remained stable from 2004 to 2011.

**Table 1:** Patient characteristics

Characteristics	Total N = 529 (%)	No primary tumor resection N = 299 (%)	Primary tumor resection N = 230 (%)	*P* a
Age (y)				
20‐39	34 (6.4)	2 (0.7)	32 (13.9)	<0.001
40‐59	219 (41.4)	119 (39.8)	100 (43.5)	
60‐79	237 (44.8)	147 (49.2)	90 (39.1)	
80+	39 (7.4)	31 (10.4)	8 (3.5)	
Race				
White	355 (67.1)	213 (71.2)	142 (61.7)	<0.001
Black	80 (15.1)	54 (18.1)	26 (11.3)	
Other	94 (17.8)	32 (10.7)	62 (27.0)	
Sex				
Male	418 (79.0)	248 (82.9)	170 (73.9)	0.011
Female	111 (21.0)	51 (17.1)	60 (26.1)	
Year				
2004‐2008	282 (53.3)	169 (56.5)	113 (49.1)	0.091
2009‐2013	247 (46.7)	130 (43.5)	117 (50.9)	
Tumor size				
<3 cm	32 (6.0)	16 (5.4)	16 (7.0)	<0.001
3‐4.9 cm	72 (13.6)	38 (12.7)	34 (14.8)	
5‐10 cm	163 (30.8)	89 (29.8)	74 (32.2)	
>10 cm	126 (23.8)	49 (16.4)	77 (33.5)	
Unknown	136 (25.7)	107 (35.8)	29 (12.6)	
Stage^b^				
IVa	152 (28.7)	61 (20.4)	91 (39.6)	<0.001
IVb	377 (71.3)	238 (79.6)	139 (60.4)	
AFP				
Negative	92 (17.4)	30 (10.0)	62 (27.0)	<0.001
Positive	295 (55.8)	178 (59.5)	117 (50.9)	
Unknown	142 (26.8)	91 (30.4)	51 (22.2)	
Primary tumor number				
Single	173 (32.7)	84 (28.1)	89 (38.7)	0.008
Multiple	159 (30.1)	88 (29.4)	71 (30.9)	
Unknown	197 (37.2)	127 (42.5)	70 (30.4)	
Vascular invasion				
No	175 (33.1)	92 (30.8)	83 (36.1)	0.218
Yes	127 (24.0)	69 (23.1)	58 (25.2)	
Unknown	227 (42.9)	138 (46.2)	89 (38.7)	
Extrahepatic extension				
No	393 (74.3)	216 (72.2)	177 (77.0)	<0.001
Yes	77 (14.6)	34 (11.4)	43 (18.7)	
Unknown	59 (11.2)	49 (16.4)	10 (4.3)	
Radiotherapy				
No	461 (87.1)	258 (86.3)	203 (88.3)	0.777
Yes	65 (12.3)	39 (13.0)	26 (11.3)	
Unknown	3 (0.6)	2 (0.7)	1 (0.4)	

a *χ* ^2^ test.

b AJCC, American Joint Committee on Cancer (7th edition).

### 3.2 PTR and its impact on prognosis

**Figure 2:**
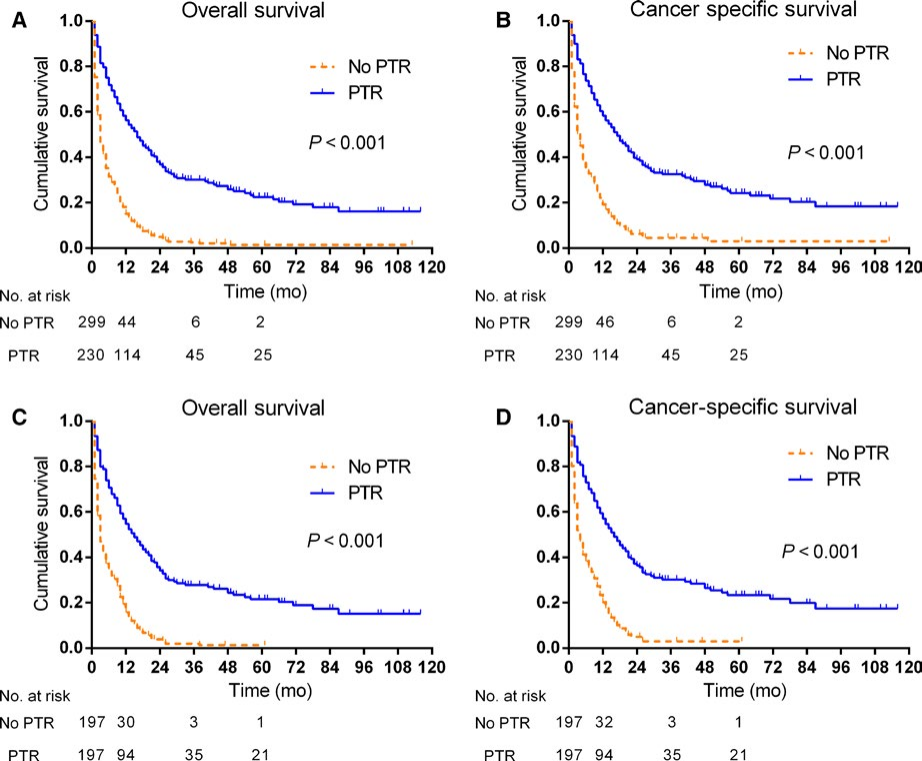
Kaplan‐Meier curves of HCC patients with resectable primary tumor but with extrahepatic metastases according to whether PTR was carried out or not in the SEER population. Both overall survival (A) and cancer‐specific survival (B) were significantly better in the PTR group (*P *<* *0.001). After propensity score matching, PTR also improved overall survival (C) and cancer‐specific survival (D) (*P *<* *0.001)

The prognostic impact of PTR was evaluated by comparing the OS and CSS between the two groups. The cumulative survival curve demonstrated the PTR group had a lower overall mortality when compared with the no‐PTR group (Figure 2A), with similar patterns for the cancer‐specific mortality (Figure 2B). The median OS and CSS time for patients without PTR were 3 months and 4 months, respectively, while they were 15 months and 17 months in the PTR group, respectively. For the no‐PTR group, the 1‐, 3‐, and 5‐year OS and CSS rates were 14.7%, 2%, and 0.6% and 15.4%, 2%, and 0.6%, respectively. By contrast, the 1‐, 3‐, and 5‐year OS and CSS rates for the PTR group were 49.5%, 19.5%, and 10.8% and 49.5%, 19.5%, and 10.8%, respectively, which were significantly higher than that in the no‐PTR group.

Multivariate survival analysis identified the factors to be significantly associated with worse OS were age beyond 80 years (HR 2.114, 95% CI 1.243‐3.594, *P *=* *0.006), huge tumors with a diameter ≥10 cm (HR 1.995, 95% CI 1.226‐3.247, *P *=* *0.005), Stage IVb (HR 1.452, 95% CI 1.156‐1.825, *P *=* *0.001), and vascular invasion (HR 1.489, 95% CI 1.147‐1.933, *P *=* *0.003). On the other hand, PTR significantly improved OS (HR 0.329, 95% CI 0.260‐0.416, *P *<* *0.001; Table 2). Similar results were obtained for CSS with poor prognostic factors to be associated with age over 80 years (HR 2.062, 95% CI 1.188‐3.578, *P *=* *0.010), huge tumors with a diameter ≥10 cm (HR 1.857, 95% CI 1.127‐3.061, *P *=* *0.015), Stage IVb (HR 1.433, 95% CI 1.132‐1.815, *P *=* *0.003), vascular invasion (HR 1.486, 95% CI 1.130‐1.954, *P *=* *0.005), and PTR (HR 0.355, 95% CI 0.279‐0.453, *P *<* *0.001; Table 2).

**Table 2:** Multivariate analysis of overall survival and cancer‐specific survival for the whole study population before and after propensity score matching

Characteristics	Overall survival				Cancer‐specific survival			
	Before matching		After matching		Before matching		After matching	
	HR (95% CI)	*P*	HR (95% CI)	*P*	HR (95% CI)	*P*	HR (95% CI)	*P*
Age (y)								
20‐39	Reference		Reference		Reference		Reference	
40‐59	1.225 (0.804‐1.867)	0.345	1.456 (0.876‐2.420)	0.147	1.211 (0.782‐1.874)	0.391	1.465 (0.868‐2.470)	0.153
60‐79	1.214 (0.796‐1.851)	0.367	1.473 (0.885‐2.452)	0.136	1.678 (0.753‐1.811)	0.488	1.418 (0.838‐2.399)	0.194
80+	**2.114 (1.243‐3.594)**	**0.006**	2.013 (0.949‐4.267)	0.068	**2.062 (1.188‐3.578)**	**0.010**	1.631 (0.709‐3.749)	0.250
Tumor size								
<3 cm	Reference		Reference		Reference		Reference	
3‐4.9 cm	1.009 (0.604‐1.688)	0.972	1.172 (0.654‐2.100)	0.594	0.979 (0.576‐1.665)	0.937	1.132 (0.621‐2.065)	0.686
5‐10 cm	1.413 (0.884‐2.260)	0.148	1.684 (0.982‐2.886)	0.058	1.297 (0.801‐2.099)	0.290	1.575 (0.900‐2.755)	0.112
>10 cm	**1.995 (1.226‐3.247)**	**0.005**	**2.310 (1.321‐4.038)**	**0.003**	**1.857 (1.127‐3.061)**	**0.015**	**2.245 (1.258‐4.006)**	**0.006**
Unknown	1.605 (0.989‐2.604)	0.055	1.797 (1.019‐3.170)	0.043	1.504 (0.918‐2.464)	0.105	1.891 (1.056‐3.388)	0.032
Stage^†^								
IVa	Reference		Reference		Reference		Reference	
IVb	**1.452 (1.156‐1.825)**	**0.001**	**1.498 (1.170‐1.919)**	**0.001**	**1.433 (1.132‐1.815)**	**0.003**	**1.439 (1.108‐1.871)**	**0.006**
AFP								
Negative	Reference		NA	NA	Reference		NA	NA
Positive	1.204 (0.909‐1.595)	0.194	NA	NA	1.236 (0.920‐1.661)	0.159	NA	NA
Unknown	0.919 (0.659‐1.281)	0.618	NA	NA	0.874 (0.620‐1.233)	0.443	NA	NA
Primary tumor number								
Single	NA	NA	Reference		NA	NA	NA	NA
Multiple	NA	NA	1.099 (0.833‐1.450)	0.503	NA	NA	NA	NA
Unknown	NA	NA	1.324 (1.005‐1.744)	0.046	NA	NA	NA	NA
Vascular invasion								
No	Reference		NA	NA	Reference		Reference	
Yes	**1.489 (1.147‐1.933)**	**0.003**	NA	NA	**1.486 (1.130‐1.954)**	**0.005**	**1.395 (1.017‐1.912)**	**0.039**
Unknown	1.363 (1.040‐1.786)	0.025	NA	NA	1.278 (1.000‐1.633)	0.050	1.346 (1.022‐1.772)	0.035
Primary tumor resection								
No	Reference		Reference		Reference		Reference	
Yes	**0.329 (0.260‐0.416)**	**<0.001**	**0.310 (0.241‐0.400)**	**<0.001**	**0.355 (0.279‐0.453)**	**<0.001**	**0.326 (0.250‐0.425)**	**<0.001**

Bold values: Statistical differences are significant.

### 3.3 Propensity score matching to adjust for patient characteristics

To minimize selection biases, propensity score matching (PSM) was used (Table S1). After matching, the patient characteristics in the PTR group were adjusted to match those of the no‐PTR group. After PSM, significant differences in the Kaplan‐Meier survival curves existed between the two groups of patients (log‐rank test, *P *<* *0.001; Figure 2C,D). The median OS time and CSS time for patients without PTR were 3 months and 4 months, respectively, while they were 15 months and 16 months in the PTR group, respectively. The 1‐, 3‐, and 5‐year OS and CSS rates in the PTR cohort were significantly higher than the no‐PTR group. On multivariate survival analysis after propensity score matching, huge tumors with a diameter ≥10 cm and Stage IVb remained poor prognostic factors of overall mortality (HR 2.310, 95% CI 1.321‐4.038, *P *=* *0.003; HR 1.498, 95% CI 1.170‐1.919, *P *=* *0.001) and cancer‐specific mortality (HR 2.245, 95% CI 1.258‐4.006, *P *=* *0.006; HR 1.439, 95% CI 1.108‐1.871, *P *=* *0.006; Table 2). In addition, multivariate analysis also identified vascular invasion as an independent poor predictor of CSS but not of OS (HR 1.395, 95% CI 1.017‐1.912, *P *=* *0.039; Table 2). However, old age beyond 80 years was not found to be an independent predictor of OS and CSS (*P *=* *0.068; *P *=* *0.250). PTR was associated with improved OS (HR 0.310, 95% CI 0.241‐0.400, *P *<* *0.001) and CSS (HR 0.326, 95% CI 0.250‐0.425, *P *<* *0.001; Table 2) in the propensity score‐matched patients.

### 3.4 Multiple imputation and sensitivity analysis

The multiple imputation procedure was applied to account for the missing data of some of the key variables in the SEER database. All the standardized mean differences were reduced after propensity score matching and multiple imputation (Table S2). Similarly, significant differences in survival curves were also observed (Figure S1). The median OS time and CSS time for the patients without PTR were 3 months and 4 months, respectively, while they were 14 months and 15 months in the PTR group, respectively. Importantly, this improvement of PTR in outcomes remained significant after sensitivity analyses using multiple imputation for overall mortality (HR 0.36, 95% CI 0.283‐0.458, *P *<* *0.001) and cancer‐specific mortality (HR 0.396, 95% CI 0.308‐0.510, *P *<* *0.001; Table S3). Vascular invasion and Stage IVb were identified as independent predictors of OS and CSS. However, sensitivity analyses showed that huge tumors with a diameter ≥10 cm were not an independent predictor of OS and CSS (HR 1.570, 95% CI 0.973‐2.534, *P *=* *0.065; HR 1.550 95% CI 0.937‐2.563, *P *=* *0.088).

### 3.5 Validating the survival benefit of PTR

**Figure 3:**
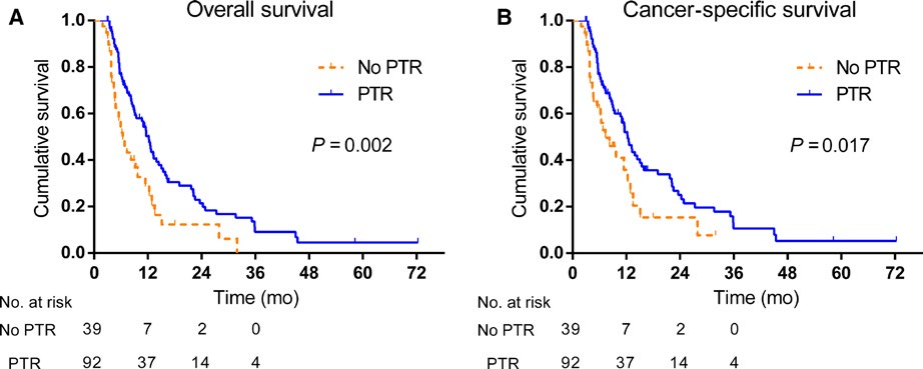
Survival curves of 131 HCC patients with resectable primary tumor but with extrahepatic metastases from the Sun Yat‐sen Memorial Hospital. The patient cohort was divided into whether PTR was carried out or not. Patients underwent PTR had a favorable survival outcome when compared with patients without PTR (OS:* P *=* *0.002; CSS:* P *=* *0.017)

Keeping the same criteria as used in the SEER cohort, 131 patients were enrolled in the external validation patient cohort from the Sun Yat‐sen Memorial Hospital. Among the 131 HCC patients with extrahepatic metastases with resectable primary tumors, 92 patients underwent PTR, whereas 39 did not. All the clinicopathological and demographic factors between the two groups of patients showed no significant difference (Table 3). On comparison of the survival curves (Figure 3), patients who underwent PTR had more favorable survival than the patients without PTR (OS: *P *=* *0.002; CSS: *P *=* *0.017). The median OS times for the patients with or without PTR were 12.2 months and 6.4 months, and the median CSS times were 12.3 months and 7.3 months, respectively. For the no‐PTR group, the 1‐, 2‐, and 3‐year OS and CSS rates were 17.9%, 5.1%, and 0% and 17.9%, 5.1%, and 0%, respectively. The corresponding OS and CSS rates for the PTR group were 40.6%, 15.4%, and 4.4% and 40.2%, 15.2%, and 4.3%, respectively. Furthermore, as in the SEER cohort, PTR was correlated in the external validation cohort with improved OS (HR 0.508, 95% CI 0.329‐0.783, *P *=* *0.002) and CSS (HR 0.568, 95% CI 0.356‐0.904, *P *=* *0.017).

**Table 3:** Patient characteristics of Sun Yat‐sen Memorial Hospital

Characteristics	Total N = 131 (%)	No primary tumor resection N = 39 (%)	Primary tumor resection N = 92 (%)	*P* a
Age (y)				
20‐39	24 (18.3)	6 (15.4)	18 (19.6)	0.744
40‐59	75 (57.3)	22 (56.4)	53 (57.6)	
60+	32 (24.4)	11 (28.2)	21 (22.8)	
Sex				
Male	113 (86.3)	31 (79.5)	82 (89.1)	0.143
Female	18 (13.7)	8 (20.5)	10 (10.9)	
Year				
2006‐2010	39 (29.8)	13 (33.3)	26 (28.3)	0.562
2011‐2015	92 (70.2)	26 (66.7)	66 (71.7)	
Tumor size (cm)				
<3	5 (3.8)	2 (5.1)	3 (3.3)	0.857
3‐4.9	13 (9.9)	3 (7.7)	10 (10.9)	
5‐10	67 (51.1)	19 (48.7)	48 (52.2)	
>10	46 (35.1)	15 (38.5)	31 (33.7)	
Stage^b^				
IVa	68 (62.6)	21 (53.9)	47 (51.1)	0.773
IVb	63 (37.4)	18 (46.1)	45 (48.9)	
AFP				
Negative	36 (27.5)	14 (35.9)	22 (23.9)	0.160
Positive	95 (72.5)	25 (64.1)	70 (76.1)	
Primary tumor number				
Single	82 (62.6)	25 (64.1)	57 (62.0)	0.816
Multiple	49 (37.4)	14 (35.9)	35 (38.0)	
Vascular invasion				
No	59 (33.1)	20 (51.3)	39 (42.4)	0.350
Yes	72 (24.0)	19 (48.7)	53 (57.6)	
Extrahepatic extension				
No	96 (73.3)	31 (79.5)	65 (70.6)	0.296
Yes	35 (26.7)	8 (20.5)	27 (29.4)	
Child‐Pugh				
A	117 (89.3)	34 (87.2)	83 (90.2)	0.607
B	14 (10.7)	5 (12.8)	9 (9.8)	
Cirrhosis CT grade				
I	90 (68.7)	24 (61.5)	66 (71.7)	0.108
II	37 (28.2)	12 (30.8)	25 (27.2)	
III	4 (3.1%)	3 (7.7)	1 (1.1)	
IV	0 (0)	0 (0)	0 (0)	
Metastases number				
Single	100 (76.3)	29 (74.4)	71 (77.2)	0.729
Multiple	31 (23.7)	10 (25.6)	21 (22.8)	

a *χ* ^2^ test.

b AJCC, American Joint Committee on Cancer (7th edition).

## 4 DISCUSSION

This study collected from the SEER registry between 2004 and 2013. Using propensity score matching and multiple imputation, PTR was shown to result in better OS and CSS in HCC patients with extrahepatic metastases. The survival benefit of PTR was then validated using an independent external validation cohort from the Sun Yat‐sen Memorial Hospital.

Several retrospective studies reported that local or regional treatment which aimed to control intrahepatic tumors resulted in better survival in HCC patients with extrahepatic metastases. A retrospective study on 277 HCC patients with extrahepatic metastases demonstrated that by applying local treatment specifically to target intrahepatic lesions to be a significant factor which affected survival of HCC patients with extrahepatic metastases.^6^ Aino and his associates showed that in patients who underwent or did not undergo treatment for primary liver cancer, the intrahepatic tumor status was an independent predictor of survival in HCC patients with extrahepatic metastases.^16^ The combined modality approach, including the use of PTR, has been shown to result in better prognosis for patients with metastatic disease in other malignancies, such as ovarian, colorectal, and renal carcinoma.^17^, ^18^, ^19^

For HCC patients with extrahepatic metastases, the BCLC guidelines recommend sorafenib to be the only palliative treatment.^2^, ^20^, ^21^ The NCCN guidelines recommend systemic therapy such as sorafenib and chemotherapy, best supportive care, and clinical trial to be conducted on these patients.^9^ PTR was not recommended by these guidelines for patients with extrahepatic metastases. However, what is very interesting in the latest version BCLC guideline is the added box under all suggested treatment options by stage which mentioned that “Effective treatments with survival benefit”.^21^ The endpoint of treatment is to increase survival. It may suggest that even if in the past surgical approach was not advised and now as long as it confers a significant survival benefit can be applied.

Partial hepatectomy is generally considered to be an effective therapy for patients with a liver tumor of any size, who had good performance status, favorable overall hepatic function (Child‐Pugh class A and selected Child‐Pugh class B), adequate liver remnant volume, and without gross vascular invasion.^9^ Interestingly, in a multicenter study, Roayaie and his associates divided 8656 HCC patients from Asia, Europe, and North America into four groups: patients who met standard resection criteria to undergo either resection (n = 718) or no resection (n = 144); and patients who did not meet standard resection criteria to undergo either resection (n = 1624) or no resection (n = 6170).^22^ Notably, resection was associated with lower mortality rates compared to embolization and other therapies for patients who did not meet the standard resection criteria but underwent resection. The authors, therefore, suggested that hepatic resection should be used more freely. Furthermore, as published studies have showed that by controlling intrahepatic HCC by hepatic resection, TACE, RFA, and radiotherapy in HCC patients with extrahepatic metastases, long‐term survival outcomes improved.^5^, ^6^, ^8^, ^23^ These studies showed that in HCC patients with extrahepatic metastases, liver failure caused by progression of intrahepatic lesions was the main cause of death.

With improvements in medical imagings, an increasing number of HCC patients with a single liver lesion with favorable residual liver volume and function but with extrahepatic metastases are diagnosed. This accounts for the increasing rate of PTR in HCC patients with extrahepatic metastases in the SEER study. Han and his associates reported that young patients less than 60 years old could be considered as a positive survival factor.^24^ In our study, old age (>60 years) was not an independent negative prognostic factor of OS and CSS after propensity score matching and multiple imputation on multivariate analysis. Although small intrahepatic tumor (diameter <3 cm) has been considered as a favorable prognostic factor,^6^ our study failed to identify it as an independent factor of OS and CSS. The possible explanations for these inconsistencies are that the treatment modalities for the intrahepatic lesions in their study were diverse (hepatic resection, TACE, RFA, and radiotherapy), and 47.3% patients had no extrahepatic metastases. These two factors differ significantly from our study. In consistence with other studies, vascular invasion and Stage IVb were identified as independent predictors of OS and CSS in our study.

Our study has several strengths. First, a large population‐based data from SEER, instead of data from a single institution, were used to avoid heterogeneity in different centers. Second, propensity score matching was used to minimize selection biases. Multiple imputation was also performed to impute missing data for significant variables. Third, an independent external validation patient cohort was used to verify results.

There are limitations of this study. First, this study is retrospective. Second, our multivariable survival analyses can only analyze common prognostic factors as SEER cannot offer other potential predictors including the criteria of selecting patients to undergo resection, the type of primary tumor resection, status of metastatic lesions, and performance status of patients for analysis.

In conclusion, this study demonstrated that PTR resulted in favorable survival outcomes for HCC patients with extrahepatic metastases. Further prospective randomized controlled trials are needed to determine the role of PTR in the treatment of HCC patients with resectable primary tumors but with extrahepatic metastases.

## CONFLICT OF INTEREST

None declared.

## References

[1] Lee HS . Management of patients with hepatocellular carcinoma and extrahepatic metastasis. Dig Dis. 2011;29:333‐338. 21829026 10.1159/000327572

[2] Bruix J , Sherman M . American Association for the Study of Liver D. Management of hepatocellular carcinoma: an update. Hepatology. 2011;53:1020‐1022. 21374666 10.1002/hep.24199 PMC3084991

[3] Kudo M , Izumi N , Kokudo N , et al. Management of hepatocellular carcinoma in Japan: Consensus‐Based Clinical Practice Guidelines proposed by the Japan Society of Hepatology (JSH) 2010 updated version. Dig Dis. 2011;29:339‐364. 21829027 10.1159/000327577

[4] Uka K , Aikata H , Takaki S , et al. Clinical features and prognosis of patients with extrahepatic metastases from hepatocellular carcinoma. World J Gastroenterol. 2007;13:414‐420. 17230611 10.3748/wjg.v13.i3.414 PMC4065897

[5] Uchino K , Tateishi R , Shiina S , et al. Hepatocellular carcinoma with extrahepatic metastasis: clinical features and prognostic factors. Cancer. 2011;117:4475‐4483. 21437884 10.1002/cncr.25960

[6] Lee JI , Kim JK , Kim Do Y , et al. Prognosis of hepatocellular carcinoma patients with extrahepatic metastasis and the controllability of intrahepatic lesions. Clin Exp Metas. 2014;31:475‐482. 10.1007/s10585-014-9641-x 24496959

[7] Yoo DJ , Kim KM , Jin YJ , et al. Clinical outcome of 251 patients with extrahepatic metastasis at initial diagnosis of hepatocellular carcinoma: does transarterial chemoembolization improve survival in these patients? J Gastroenterol Hepatol. 2011;26:145‐154. 21175808 10.1111/j.1440-1746.2010.06341.x

[8] Jung SM , Jang JW , You CR , et al. Role of intrahepatic tumor control in the prognosis of patients with hepatocellular carcinoma and extrahepatic metastases. J Gastroenterol Hepatol. 2012;27:684‐689. 21916984 10.1111/j.1440-1746.2011.06917.x

[9] Benson AB , D'Angelica M , Abbott D . NCCN Guidelines Insights: Hepatobiliary Cancers, Version 1.2017. J Natl Compr Canc Netw. 2017;15(5):563‐573. 28476736 10.6004/jnccn.2017.0059 PMC5557008

[10] Utsunomiya T , Shimada M , Kudo M , et al. Nationwide study of 4741 patients with non‐B non‐C hepatocellular carcinoma with special reference to the therapeutic impact. Ann Surg. 2014;259:336‐345. 23673768 10.1097/SLA.0b013e31829291e9

[11] Klebanoff MA , Cole SR . Use of multiple imputation in the epidemiologic literature. Am J Epidemiol. 2008;168:355‐357. 18591202 10.1093/aje/kwn071 PMC2561989

[12] Janssen KJ , Donders AR , Harrell FE Jr , et al. Missing covariate data in medical research: to impute is better than to ignore. J Clin Epidemiol. 2010;63:721‐727. 20338724 10.1016/j.jclinepi.2009.12.008

[13] Liao SG , Lin Y , Kang DD , et al. Missing value imputation in high‐dimensional phenomic data: imputable or not, and how? BMC Bioinformatics. 2014;15:346. 25371041 10.1186/s12859-014-0346-6 PMC4228077

[14] Little RR . Statistical Analysis with Missing Data (Second Edition). New York, NY: John Wiley & Sons; 2002.

[15] Team RC . A Language and Environment for Statistical Computing. Vienna, Austria: R Foundation for Statistical Computing; 2014.

[16] Aino H , Sumie S , Niizeki T , et al. Clinical characteristics and prognostic factors for advanced hepatocellular carcinoma with extrahepatic metastasis. Mol Clin Oncol. 2014;2:393‐398. 24772306 10.3892/mco.2014.259 PMC3999133

[17] Dauplat J , Le Bouedec G , Pomel C , Scherer C . Cytoreductive surgery for advanced stages of ovarian cancer. Semin Surg Oncol. 2000;19:42‐48. 10883023 10.1002/1098-2388(200007/08)19:1<42::aid-ssu7>3.0.co;2-m

[18] Rosen SA , Buell JF , Yoshida A , et al. Initial presentation with stage IV colorectal cancer: how aggressive should we be? Arch Surg. 2000;135:530‐534; discussion 4‐5. 10807276 10.1001/archsurg.135.5.530

[19] Flanigan RC , Salmon SE , Blumenstein BA , et al. Nephrectomy followed by interferon alfa‐2b compared with interferon alfa‐2b alone for metastatic renal‐cell cancer. N Engl J Med. 2001;345:1655‐1659. 11759643 10.1056/NEJMoa003013

[20] Forner A , Llovet JM , Bruix J . Hepatocellular carcinoma. Lancet. 2012;379:1245‐1255. 22353262 10.1016/S0140-6736(11)61347-0 PMC13537732

[21] Bruix J , Reig M , Sherman M . Evidence‐based diagnosis, staging, and treatment of patients with hepatocellular carcinoma. Gastroenterology. 2016;150:835‐853. 26795574 10.1053/j.gastro.2015.12.041

[22] Roayaie S , Jibara G , Tabrizian P , et al. The role of hepatic resection in the treatment of hepatocellular cancer. Hepatology. 2015;62:440‐451. 25678263 10.1002/hep.27745

[23] Jun L , Zhenlin Y , Renyan G , et al. Independent factors and predictive score for extrahepatic metastasis of hepatocellular carcinoma following curative hepatectomy. Oncologist. 2012;17:963‐969. 22653882 10.1634/theoncologist.2011-0447 PMC3399653

[24] Han JH , Kim DG , Park JC , Chung HT , Paek SH , Chung YS . Little response of cerebral metastasis from hepatocellular carcinoma to any treatments. J Korean Neurosurg Soc. 2010;47:325‐331. 20539790 10.3340/jkns.2010.47.5.325 PMC2883051

