# Supplementary material for: The impact of liver resection on survival outcomes of hepatocellular carcinoma patients with extrahepatic metastases: A propensity score matching study
Source: Cancer Med. 2018 Aug 16;7(9):4475–84. doi: 10.1002/cam4.1738 (PMC6143947; doi:10.1002/cam4.1738)
Supplement: Supplementary file 2 [file CAM4-7-4475-s002.docx]

Supplemental Table 1 Characteristics and standardised mean differences of covariates among patients underwent primary tumor resection (PTR) or not before and after propensity-score matching.

| Characteristics | Before matching | | | |  | | After matching | | |
| --- | --- | --- | --- | --- | --- | --- | --- | --- | --- |
|  | No PTR^*^  (%) | PTR  (%) | | Std. mean difference | |  | No PTR (%) | PTR (%) | Std. mean difference |
| Age |  |  |  | |  | |  |  |  |
| 20-39 yr | 0.7 | 13.9 | 0.527 | |  | | 1.0 | 9.6 | 0.391 |
| 40-59 yr | 39.8 | 43.5 | 0.075 | |  | | 49.2 | 41.6 | -0.153 |
| 60-79 yr | 49.2 | 39.1 | -0.203 | |  | | 46.2 | 44.7 | -0.031 |
| 80+ yr | 10.4 | 3.5 | -0.274 | |  | | 3.6 | 4.1 | 0.027 |
| Race |  |  |  | |  | |  |  |  |
| White | 71.2 | 61.7 | -0.202 | |  | | 64.0 | 66.0 | 0.043 |
| Black | 18.1 | 11.3 | -0.192 | |  | | 20.8 | 11.7 | -0.250 |
| Other | 10.7 | 27.0 | 0.425 | |  | | 15.2 | 22.3 | 0.183 |
| Sex |  |  |  | |  | |  |  |  |
| Male | 82.9 | 73.9 | -0.221 | |  | | 81.7 | 78.2 | -0.089 |
| Female | 17.1 | 26.1 | 0.221 | |  | | 18.3 | 21.8 | 0.089 |
| Year |  |  |  | |  | |  |  |  |
| 2004-2008 | 56.5 | 49.1 | -0.148 | |  | | 48.2 | 49.7 | 0.030 |
| 2009-2013 | 43.5 | 50.9 | 0.148 | |  | | 51.8 | 50.3 | -0.030 |
| Tumor Size |  |  |  | |  | |  |  |  |
| <3cm | 5.4 | 7.0 | 0.067 | |  | | 7.1 | 5.1 | -0.085 |
| 3-4.9cm | 12.7 | 14.8 | 0.060 | |  | | 15.2 | 15.7 | 0.014 |
| 5-10cm | 29.8 | 32.2 | 0.052 | |  | | 35.0 | 29.9 | -0.109 |
| >10cm | 16.4 | 33.5 | 0.403 | |  | | 19.3 | 34.5 | 0.349 |
| Unknown | 35.8 | 12.6 | -0.562 | |  | | 23.4 | 14.7 | -0.221 |
| Stage^†^ |  |  |  | |  | |  |  |  |
| IVa | 20.4 | 39.6 | 0.428 | |  | | 27.4 | 35.5 | 0.176 |
| IVb | 79.6 | 60.4 | -0.428 | |  | | 72.6 | 64.5 | -0.176 |
| AFP |  |  |  | |  | |  |  |  |
| Negative | 10.0 | 27.0 | 0.447 | |  | | 10.2 | 26.9 | 0.442 |
| Positive | 59.5 | 50.9 | -0.175 | |  | | 66.0 | 50.3 | -0.323 |
| Unknown | 30.4 | 22.2 | -0.188 | |  | | 23.9 | 22.8 | -0.024 |
| Primary Tumor Number |  |  |  | |  | |  |  |  |
| Single | 28.1 | 38.7 | 0.226 | |  | | 32.5 | 37.6 | 0.107 |
| Multiple | 29.4 | 30.9 | 0.031 | |  | | 22.8 | 30.5 | -0.087 |
| Unknown | 42.5 | 30.4 | -0.252 | |  | | 41.6 | 32.0 | -0.022 |
| Vascular Invasion |  |  |  | |  | |  |  |  |
| No | 30.8 | 36.1 | 0.113 | |  | | 35.5 | 34.0 | -0.032 |
| Yes | 23.1 | 25.2 | 0.050 | |  | | 22.8 | 26.9 | 0.094 |
| Unknown | 46.2 | 38.7 | -0.151 | |  | | 41.6 | 39.1 | -0.052 |
| Extrahepatic Extension |  |  |  | |  | |  |  |  |
| No | 72.2 | 77.0 | 0.108 | |  | | 82.2 | 75.6 | -0.162 |
| Yes | 11.4 | 18.7 | 0.206 | |  | | 9.6 | 19.3 | 0.277 |
| Uknown | 16.4 | 4.3 | -0.403 | |  | | 8.1 | 5.1 | -0.123 |
| Radiotherapy |  |  |  | |  | |  |  |  |
| No | 86.3 | 88.3 | 0.059 | |  | | 86.8 | 87.8 | 0.031 |
| Yes | 13.0 | 11.3 | -0.053 | |  | | 12.7 | 11.7 | -0.031 |
| Unknown | 0.7 | 0.4 | -0.032 | |  | | 0.5 | 0.5 | 0.000 |

*PTR, Primary Tumor Resection.

† AJCC, American Joint Committee on Cancer (7^th^ edition).
