# Supplementary material for: The impact of liver resection on survival outcomes of hepatocellular carcinoma patients with extrahepatic metastases: A propensity score matching study
Source: Cancer Med. 2018 Aug 16;7(9):4475–84. doi: 10.1002/cam4.1738 (PMC6143947; doi:10.1002/cam4.1738)
Supplement: Supplementary file 3 [file CAM4-7-4475-s003.docx]

Supplemental Table 2 Characteristics and standardised mean differences of covariates among patients underwent primary tumor resection (PTR) or not before and after propensity-score matching and multiple imputation.

| Characteristics | Before matching | | | |  | | After matching | | |
| --- | --- | --- | --- | --- | --- | --- | --- | --- | --- |
|  | No PTR^*^  (%) | PTR  (%) | | Std. mean difference | |  | No PTR (%) | PTR (%) | Std. mean difference |
| Age |  |  |  | |  | |  |  |  |
| 20-39 yr | 0.7 | 13.9 | 0.527 | |  | | 1.0 | 9.4 | 0.382 |
| 40-59 yr | 39.8 | 43.5 | 0.075 | |  | | 50.0 | 42.2 | -0.157 |
| 60-79 yr | 49.2 | 39.1 | -0.203 | |  | | 46.4 | 44.3 | -0.042 |
| 80+ yr | 10.4 | 3.5 | -0.274 | |  | | 2.6 | 4.2 | 0.086 |
| Race |  |  |  | |  | |  |  |  |
| White | 71.2 | 61.7 | -0.202 | |  | | 62.5 | 63.0 | 0.011 |
| Black | 18.1 | 11.3 | -0.192 | |  | | 23.4 | 10.9 | -0.336 |
| Other | 10.7 | 27.0 | 0.425 | |  | | 14.1 | 26.0 | 0.303 |
| Sex |  |  |  | |  | |  |  |  |
| Male | 82.9 | 73.9 | -0.221 | |  | | 81.3 | 77.1 | -0.103 |
| Female | 17.1 | 26.1 | 0.221 | |  | | 18.8 | 22.9 | 0.103 |
| Year |  |  |  | |  | |  |  |  |
| 2004-2008 | 56.5 | 49.1 | -0.148 | |  | | 46.4 | 51.0 | 0.094 |
| 2009-2013 | 43.5 | 50.9 | 0.148 | |  | | 53.6 | 49.0 | -0.094 |
| Tumor Size |  |  |  | |  | |  |  |  |
| <3cm | 7.4 | 7.0 | -0.016 | |  | | 8.9 | 8.3 | -0.019 |
| 3-4.9cm | 16.1 | 17.8 | 0.047 | |  | | 14.1 | 18.8 | 0.127 |
| 5-10cm | 50.5 | 36.5 | -0.285 | |  | | 45.8 | 35.4 | -0.213 |
| >10cm | 26.1 | 38.7 | 0.272 | |  | | 31.3 | 37.5 | 0.132 |
| Stage^†^ |  |  |  | |  | |  |  |  |
| IVa | 20.4 | 39.6 | 0.428 | |  | | 27.4 | 35.5 | 0.176 |
| IVb | 79.6 | 60.4 | -0.428 | |  | | 72.6 | 64.5 | -0.176 |
| AFP |  |  |  | |  | |  |  |  |
| Negative | 14.0 | 35.2 | 0.507 | |  | | 30.2 | 34.4 | 0.089 |
| Positive | 86.0 | 64.8 | -0.507 | |  | | 69.8 | 65.6 | -0.089 |
| Primary Tumor Number |  |  |  | |  | |  |  |  |
| Single | 39.8 | 46.1 | 0.127 | |  | | 44.3 | 45.3 | 0.021 |
| Multiple | 60.2 | 53.9 | -0.127 | |  | | 55.7 | 54.7 | -0.021 |
| Vascular Invasion |  |  |  | |  | |  |  |  |
| No | 48.8 | 45.2 | 0.072 | |  | | 45.3 | 46.4 | 0.021 |
| Yes | 51.2 | 54.8 | -0.072 | |  | | 54.7 | 53.6 | -0.021 |
| Extrahepatic Extension |  |  |  | |  | |  |  |  |
| No | 87.0 | 80.9 | -0.166 | |  | | 84.9 | 83.9 | -0.029 |
| Yes | 13.0 | 19.1 | 0.166 | |  | | 15.1 | 16.1 | 0.029 |
| Radiotherapy |  |  |  | |  | |  |  |  |
| No | 87.0 | 88.7 | 0.053 | |  | | 87.0 | 88.5 | 0.048 |
| Yes | 13.0 | 11.3 | -0.053 | |  | | 13.0 | 11.5 | -0.048 |

*PTR, Primary Tumor Resection.

† AJCC, American Joint Committee on Cancer (7^th^ edition).
