# Supplementary material for: The impact of liver resection on survival outcomes of hepatocellular carcinoma patients with extrahepatic metastases: A propensity score matching study
Source: Cancer Med. 2018 Aug 16;7(9):4475–84. doi: 10.1002/cam4.1738 (PMC6143947; doi:10.1002/cam4.1738)
Supplement: Supplementary file 4 [file CAM4-7-4475-s004.docx]

Supplemental Table 3 Multivariate analysis of overall survival and cancer-specific survival for the whole study population before and after propensity-score matching and multiple imputation.

| Characteristics | Overall Survival | | | | |  | Cancer-specific Survival | | | | |
| --- | --- | --- | --- | --- | --- | --- | --- | --- | --- | --- | --- |
|  | **Before matching** | |  | **After matching** | |  | **Before matching** | |  | **After matching** | |
|  | HR (95% CI) | *P* |  | HR (95% CI) | *P* |  | HR (95% CI) | *P* |  | HR (95% CI) | *P* |
| Age |  |  |  |  |  |  |  |  |  |  |  |
| 20-39 yr | Reference |  |  | Reference |  |  | Reference |  |  | Reference |  |
| 40-59 yr | 1.206 (0.796-1.829) | 0.377 |  | 1.565 (0.930-2.632) | 0.091 |  | 1.234 (0.801-1.900) | 0.341 |  | 1.588 (0.928-2.717) | 0.091 |
| 60-79 yr | 1.171 (0.771-1.778) | 0.460 |  | 1.440 (0.856-2.422) | 0.169 |  | 1.155 (0.748-1.784) | 0.515 |  | 1.351 (0.788-2.315) | 0.274 |
| 80+ yr | **1.714 (1.017-2.888)** | **0.043** |  | 2.128 (0.958-4.727) | 0.064 |  | **1.742 (1.012-3.001)** | **0.045** |  | 1.818 (0.765-4.321) | 0.176 |
| Tumor Size |  |  |  |  |  |  |  |  |  |  |  |
| <3cm | Reference |  |  | Reference |  |  | Reference |  |  | Reference |  |
| 3-4.9cm | 1.000 (0.635-1.573) | 0.998 |  | 0.933 (0.553-4.576) | 0.796 |  | 0.954 (0.594-1.532) | 0.845 |  | 0.884 (0.508-1.537) | 0.662 |
| 5-10cm | 1.327 (0.880-2.000) | 0.177 |  | 1.326 (0.832-2.113) | 0.235 |  | 1.317 (0.860-2.018) | 0.206 |  | 1.303 (0.799-2.126) | 0.289 |
| >10cm | **1.587 (1.038-2.428)** | **0.033** |  | **1.570 (0.973-2.534)** | **0.065** |  | **1.559 (1.002-2.426)** | **0.049** |  | 1.550 (0.937-2.563) | 0.088 |
| Stage^†^ |  |  |  |  |  |  |  |  |  |  |  |
| IVa | Reference |  |  | Reference |  |  | Reference |  |  | Reference |  |
| IVb | **1.466 (1.175-1.830)** | **0.001** |  | **1.429 (1.113-1.834)** | **0.005** |  | **1.491 (1.182-1.882)** | **0.001** |  | **1.407 (1.081-1.832)** | **0.011** |
| Vascular invasion |  |  |  |  |  |  |  |  |  |  |  |
| No | Reference |  |  | Reference |  |  | Reference |  |  | Reference |  |
| Yes | **1.385 (1.142-1.678)** | **0.001** |  | **1.464 (1.163-1.843)** | **0.001** |  | **1.367 (1.118-1.673)** | **0.002** |  | **1.430 (1.121-1.825)** | **0.004** |
| Primary Tumor Resection |  |  |  |  |  |  |  |  |  |  |  |
| No | Reference |  |  | Reference |  |  | Reference |  |  | Reference |  |
| Yes | **0.338 (0.272-0.421)** | **<0.001** |  | **0.360 (0.283-0.458)** | **<0.001** |  | **0.357 (0.284-0.449)** | **<0.001** |  | **0.396 (0.308-0.510)** | **<0.001** |
